# Global malaria connectivity through air travel

**DOI:** 10.1186/1475-2875-12-269

**Published:** 2013-08-02

**Authors:** Zhuojie Huang, Andrew J Tatem

**Affiliations:** 1 Center for Infectious Disease Dynamics, Pennsylvania State University, University Park, PA, USA; 2 Department of Biology, Pennsylvania State University, PA, USA; 3 Department of Geography, University of Florida, Gainesville, FL, USA; 4 Emerging Pathogens Institute, University of Florida, Gainesville, FL, USA; 5 Department of Geography and Environment, University of Southampton, Highfield, Southampton, UK; 6 Fogarty International Center, National Institutes of Health, Bethesda, USA

## Abstract

**Background:**

Air travel has expanded at an unprecedented rate and continues to do so. Its effects have been seen on malaria in rates of imported cases, local outbreaks in non-endemic areas and the global spread of drug resistance. With elimination and global eradication back on the agenda, changing levels and compositions of imported malaria in malaria-free countries, and the threat of artemisinin resistance spreading from Southeast Asia, there is a need to better understand how the modern flow of air passengers connects each *Plasmodium falciparum-* and *Plasmodium vivax*-endemic region to the rest of the world.

**Methods:**

Recently constructed global *P. falciparum* and *P.vivax* malaria risk maps, along with data on flight schedules and modelled passenger flows across the air network, were combined to describe and quantify global malaria connectivity through air travel. Network analysis approaches were then utilized to describe and quantify the patterns that exist in passenger flows weighted by malaria prevalence. Finally, the connectivity within and to the Southeast Asia region where the threat of imported artemisinin resistance arising is highest, was examined to highlight risk routes for its spread.

**Results:**

The analyses demonstrate the substantial connectivity that now exists between and from malaria-endemic regions through air travel. While the air network provides connections to previously isolated malarious regions, it is clear that great variations exist, with significant regional communities of airports connected by higher rates of flow standing out. The structures of these communities are often not geographically coherent, with historical, economic and cultural ties evident, and variations between *P. falciparum* and *P. vivax* clear. Moreover, results highlight how well connected the malaria-endemic areas of Africa are now to Southeast Asia, illustrating the many possible routes that artemisinin-resistant strains could take.

**Discussion:**

The continuing growth in air travel is playing an important role in the global epidemiology of malaria, with the endemic world becoming increasingly connected to both malaria-free areas and other endemic regions. The research presented here provides an initial effort to quantify and analyse the connectivity that exists across the malaria-endemic world through air travel, and provide a basic assessment of the risks it results in for movement of infections.

## Background

The worldwide air travel network has expanded at an exceptional rate over the past century. International passenger numbers are projected to rise from 1.11 billion in 2011 to 1.45 billion by 2016, with an annual growth rate of 5.3% [1]. Today, there are 35,000 direct scheduled routes on the air travel network, with 865 new routes established in 2011 [2]. Malaria-endemic areas are more connected to the rest of the world than at any time in history, with the disease able to travel at speeds of 600 miles per hour within infected passengers. The growth of the air travel network results in substantial concerns and challenges to the global health system, with a need to place more emphasis on evidence-driven surveillance and reporting that incorporates spatial and network information [3-6].

Rising rates of travel between malaria-free and -endemic countries have led to general patterns of increased rates of imported malaria over recent decades [7-10]. Due to infrequent encounters [3,9], imported cases can challenge health systems in non-endemic countries, with difficulties in diagnosis [10], misdiagnosis and delays in treatment [11,12], as well as significant treatment expenses [13]. Further, flights may bring infected vectors, resulting in “airport malaria”, where patients who do not have a foreign travel history become infected through being bitten in the vicinity of international airports [14-17]. Patterns in imported cases and airport malaria have been shown to be related to a combination of the numbers of travellers and the malaria risk at the destination [3,16], and these relationships will continue to evolve as new routes become established.

The flow of people via air travel between endemic areas may increase the risks of re-emergence or resurgence [18] in previously malaria free or low transmission areas [19]. The autochthonous malaria outbreaks in Virginia in 2002 [20], Florida in 2003 [21] and Greece in 2011 [22], for example, demonstrate the continued risks of local outbreaks following reintroduction through air travel, though such occurrences are rare [23]. Further, the examples of malaria resurgence in island nations, such as Sri Lanka [24], Mauritius [25] and Madagascar [26], after control measures were relaxed reinforce the importance of vigilance and robust surveillance in terms of human movement in pre and post-elimination periods [18]. Identifying the risks of malaria movement through the air travel network can provide an evidence base through which public health practitioners and strategic planners can be informed about potential malaria influxes and their origins [3,27].

Meanwhile, growing concerns have been raised about the possible spread of artemisinin resistance from the Greater Mekong subregion in Southeast Asia to other endemic areas. Recent research has highlighted increasing numbers of patients showing slow parasite clearance rates following treatment with artemisinin-based drugs in the Cambodia-Thailand border and Thailand-Myanmar border regions [28-30]. Tremendous health and socio-economic costs occurred when chloroquine-resistant parasites arrived in sub-Saharan Africa from Southeast Asia and spread across the continent [31,32]. Similarly, sulphadoxine and pyrimethamine resistance emerged in Asia and spread to Africa [33,34]. The WHO reports that there is already “at least one study with a high treatment failure rate (≥10%) reported from six of the 23 African countries that have adopted artesunate-amodiaquine compound” [35], and fear remains over the spread of artemisinin resistance from Southeast Asia to Africa, that could undermine current control and elimination efforts, with no alternative drugs coming in the foreseeable future.

Rates of imported malaria, risks of resurgence and the spread of drug resistance are all today influenced by how the global air travel network connects up the malaria-endemic regions of the world, and the numbers of passengers moving along it. Here, recently constructed global *Plasmodium falciparum* and *Plasmodium vivax* malaria prevalence maps are combined with data on modelled passenger flows across the air network, to describe and quantify global malaria connectivity through air travel in 2010. Weighted network analysis statistics are derived to examine: (i) which regions show greatest connectivity to *P. falciparum* and *P. vivax* malaria-endemic zones; (ii) where the largest estimated passenger flows from endemic areas occur; (iii) which regions form ‘communities’, whereby malaria infection flows within them are likely to be larger than between communities, and finally, (iv) where the threat of imported artemisinin resistance is highest via air traffic, and the possible risk routes for the spread of resistance within and from Southeast Asia.

## Methods

### Airport locations, flight routes and passenger flow matrix

Information on the longitude, latitude, city name and airport code for a total of 1,449 airports which serve cities with more than 100,000 people, and a modelled ‘actual’ traffic flow (i.e. number of passengers travelling between each location and every other, irrespective of stopovers) connectivity list with 644,406 routes amongst these airports were obtained [6,36,37]. This list documented, for each origin and final travel destination, the estimated number of passengers taking this route [36] regarding the hub-and-spoke structure of the air travel network [38]. A connectivity matrix was then created from the connectivity list, quantifying the volumes and the directionalities of the passenger flows between two airports. Within this passenger flow matrix, 23,785 origin–destination pairs were connected by direct flights between two airports, 291,745 pairs were connected by routes involving one-stop and 328,876 pairs required two-stops to connect. The travel volumes on the routes were modelled based primarily on publicly available datasets under a generalized linear model framework. Full model details are provided in Huang *et al.*[36], but in brief, to construct the matrix, topological characteristics of the air travel network, city population, and local area GDP, amongst others, were utilized as covariates. Actual travel volumes for training and validation were extracted and assembled from various transportation organizations in the USA, Canada and the European Union. A log linear model controlling for random effects on origin, destination and the airport hierarchy was then built to predict passenger flows on the network. The model outperformed existing air travel passenger flow models in terms of prediction accuracy [36].

### Malaria distribution

Global *P. falciparum* and *P. vivax* prevalence maps were obtained from the Malaria Atlas Project [39] and the methods behind their construction are presented in Gething *et al.*[40,41]. In brief, 22,212 community prevalence surveys were used in combination with model-based geostatistical methods to map the prevalence of *P. falciparum* globally in 2010 within limits of transmission defined by annual parasite incidence and satellite covariate data. Similarly, 9,970 geocoded *P. vivax* parasite rate (*P. vivax* PR) surveys collected between 1985 and 2010 were utilized in a spatiotemporal Bayesian model-based geostatistical approach to map endemicity, under the restrictions of a mask of the stable/unstable endemicity [41] and information on the prevalence of the Duffy blood group [42]. Distributions of *Plasmodium ovale, Plasmodium malariae or Plasmodium knowlesi* are not included here, since similar datasets on their distributions do not yet exist. Also, the seasonal climatic constraints that affect the transmission of *P.falciparum* and *P.vivax* are not included here, but models of each [43] will be included in future work.

### Weighted network analysis and community detection

Malaria prevalence can vary greatly in the region around airports and the cities they serve, and travellers taking flights from a specific airport may reside many kilometres from the airports in higher transmission areas than found in the vicinity of the airport. Thus, simply assigning the predicted prevalence from the malaria maps at the location of each airport could underestimate the risk and rate of infection exportation at the airport in question and underrepresent its contribution to global malaria connectivity. Therefore, following Huang *et al.*[6], local accessibility to each airport was considered by assuming that passengers would travel less than 50 km with a travel time less than two hours to access an airport to take a flight. Under this assumption, the *P. falciparum* and the *P. vivax* malaria prevalence assigned to an airport were obtained as the maximum prevalence from the malaria maps within a mask of 50 km and two-hour travel time (Additional file 1), in which the mask was generated using a global travel time map [44]. This choice of the maximum value was created for a ‘worst case scenario’ assessment, but for most of the analyses conducted here, relative differences between airports were assessed or presented, thus making the choice of, for example, maximum, minimum or mean, irrelevant in most cases. Likewise, an indicator that defines whether an airport is located in the stable/unstable endemic zone was created according to the same mask, in which the indicator defines whether the majority area of the mask is located in the stable/unstable zone. Additional file 1 shows this travel time/distance mask with the global travel time map.

The above approach ensured that each airport had an assignment of a *P. falciparum* and *P. vivax* prevalence rate (or unstable/malaria free), which could then be used as a weighting applied to the passenger flow estimates to derive relative ‘malaria flow’ indices for each route, that could be compared to other routes across the global network to analyse malaria connectivity. Thus, *P. falciparum* and *P. vivax* flows were calculated on each route (either direct, one-stop or two-stops) as origin prevalence * estimated passenger volume, to produce *P. falciparum* and *P. vivax* malaria networks.

A group of weighted centrality analyses and network community partition analyses were performed on the malaria networks to quantify features of global malaria connectivity. First, the in-strength and out-strength of each connection was calculated as the sum of incoming and outgoing malaria flow via all possible connections (direct flight, one-stop or two-stops) as follows:

(1) $$s_{i}=\sum_{j=1}^{N}a_{\mathrm{ij}}w_{\mathrm{ij}}$$

in which *a*_*ij*_ is the airport adjacency matrix (in a binary form) and *w*_*ij*_ is the weighted malaria flow. This metric estimates the total weight of malaria flows that airports send and receive.

Following this, weighted “betweenness” analyses were performed on the malaria flow matrices. Betweenness centrality measures the number of shortest paths going through a specific vertex [45]. In a weighted network, betweenness centrality is a useful local measure of the load placed on the given node in the network as well as the node’s importance to the network other than just connectivity [46]. It is often used in transport network analysis to provide an approximation of the traffic handled by the vertices [47]. Thus, here it provides an indication of the status of each airport as a ‘malaria hub’ through its importance in the global flow of malaria infections via air travel - i.e., a measure of how many infections likely pass through each airport each year, relative to other airports, and how likely an airport would route and spread malaria infections onward. The betweenness centrality is calculated as:

(2) $$C_{B}\left(v\right)=\sum_{s\neq v\neq t}\frac{\delta_{\mathrm{st}}\left(v\right)}{\delta_{\mathrm{st}}}$$

in which *δ*_*st*_ is the total number of shortest paths from node s to node t and *δ*_*st*_(*v*) is the number of those paths that pass through v. Note that on the weighted *P. falciparum/P. vivax* networks, the distance between the two nodes s and t is defined by the sum of *P. falciparum* flows or *P. vivax* flows as the edge weight on this path under a classical Dijkstra shortest-path calculation framework [48]. A normalized betweenness was used as

(3) $$C_{\mathrm{Bnorm}}\left(v\right)=\frac{C_{B}\left(v\right)}{\sum C_{B}\left(v\right)/n}$$

where n was the number of nodes (airports) in the air travel network [47].

Communities in a network reflect the partition of nodes that are densely connected and separated from the other nodes in the network, thus these nodes “probably share common properties and/or play similar roles within the graph” [49]. By mapping communities on the malaria networks defined here, groups of airports that show strong links in terms of likely movements of infections were identified. This potentially has utility in terms of providing evidence upon which regional surveillance strategies can be designed [50,51]. Newman and Girvan [52] define a modularity score which measures the quality of network partitions as:

(4) $$Q=\frac{1}{2m}\sum_{i,j}\left[W_{\mathrm{ij}}-\frac{k_{i}k_{j}}{2m}\right]\delta \left(c_{i},c_{j}\right)$$

in which, *w*_*ij*_ represents the weight of the edge between i and j (here these are the *P. falciparum* and *P. vivax* flow matrices), *k*_*i*_ = ∑ _*j*_*W*_*ij*_ is the sum of the weights of the connections attached to airport i, *c*_*i*_ is the community to which airport i is assigned; *δ*(*c*_*i*_*,c*_*j*_) is 1 if *c*_*i*_ = *c*_*j*_, otherwise $m=\frac{1}{2}\sum W_{\mathrm{ij}}$.

A multilevel algorithm for community detection [53] was implemented. This method utilizes an iterative approach that merges communities to maximize the modularity score: Firstly, modularity is optimized by allowing only local changes of communities; secondly, the established communities are combined together to construct a new network. These two passes are repeated iteratively until no increase of modularity is possible. The number of communities returned by this algorithm yields the maximum modularity score.

Afterward, a simple Wilcoxon rank-sum test [54] was performed on the differences between “internal” and “external” degrees of a community in order to test whether the establishment of communities was significant. Air connections were defined within a community as “internal” and the connections connecting the airports of a community with the rest of the network as “external”. The null hypothesis of this test was that there was no difference between the number of internal and external routes incident to an airport of the community.

## Results

The results of the global malaria connectivity analyses are presented in two sections: (i) analyses focussed on the connection of endemic malaria regions to each other and to malaria-free areas, that has particular relevance to imported malaria and malaria resurgence and re-emergence; and, (ii) analyses examining the connections between Southeast Asia and the rest of the malaria-endemic world, which are relevant to the spread of artemisinin resistance.

### Connectivity within endemic areas and to non-endemic areas

**Figure 1:**
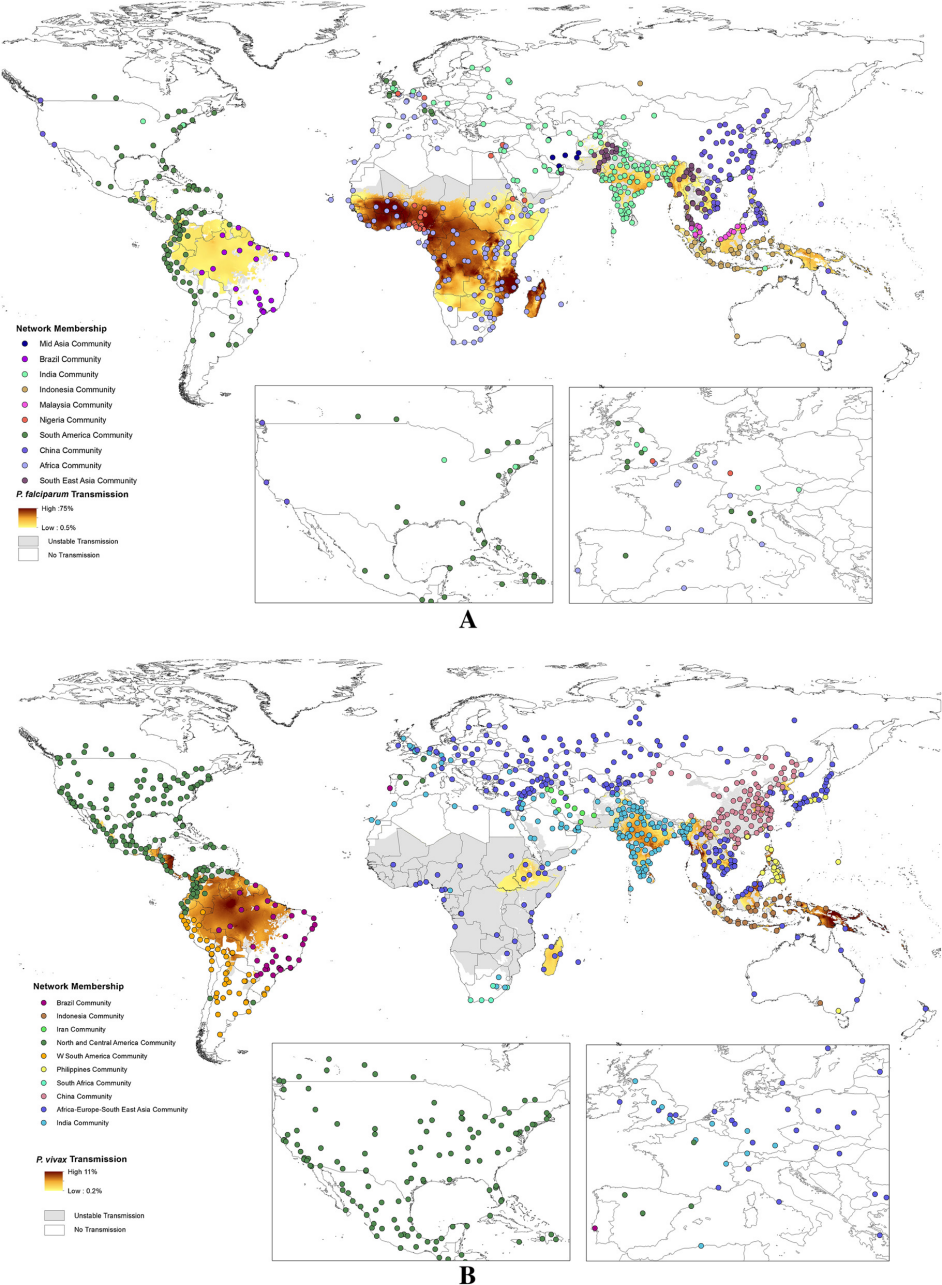
**Spatial distribution of *Plasmodium falciparum/Plasmodium vivax *network communities overlaid on *Plasmodium falciparum/Plasmodium vivax *prevalence maps. A)***P. falciparum* multilevel membership. **B)***P. vivax* multilevel membership. These two maps show only airports that have direct connections from endemic to non-endemic areas, though full origin–destination flow estimates were used in calculations. The inset maps present close-up views of the USA and western Europe. Airports with the same community membership (indicated by the same colour) display stronger links in terms of likely movements of infections between them than to airports in other communities. Note that in the *P. vivax* map, communities with less than ten airports are not shown.

Figure 1 shows the results of regional community structure analyses based on traffic flow data overlaid on the *P. falciparum/P. vivax* endemicity and stable/unstable transmission limits maps. The Wilcoxon test results show that the internal degrees for the airports within all communities are significantly different from the external degrees, with p values of <0.01, thus the community partitions shown are significant. The maps highlight those countries that form communities linked by high levels of traffic scaled by *P. falciparum/P. vivax* prevalence at their origin endemic area. Additional file 2 describes similar analyses based solely on the travel network data from Huang *et al.*[36]. The communities detected reflect the architecture of the air network, and how this relates to malaria endemicity around the world. Geographical contiguity is clearly evident, as traffic levels on shorter distance routes are generally higher than on longer distance routes, but interesting patterns relating to historical ties emerge. For instance, for *P. falciparum*, London forms part of the Nigeria community, but Paris shows stronger ties to the remainder of sub-Saharan Africa. These connections are often reflected in imported malaria statistics, with Nigeria being the main source of *P. falciparum* cases seen in the UK, but for France, the French-speaking African countries are the main origin. Similarly, UK airports also form part of the India/Bangladesh community, where historical ties exist, resulting in significant travel between the two regions, and consequent *P. falciparum* and *P. vivax* malaria importation to the UK. Ties also exist between the western USA and East Asia, which form a single *P. falciparum* community (Figure 1A). Additional file 3 shows a community detection analysis for airports with direct connections, one-transfer connections and two-transfer connections from malaria-endemic areas.

To examine directional and net potential movements of people and parasites between airports in different countries, the international route weightings were summed up to identify possible “source” and “sink” airports of malaria infections (Additional file 4). Here, the weights of all possible incoming flows for airports in the non-endemic areas, and the weights of all possible outgoing flows from airports in endemic areas were summed up to define “vertex strengths” of importation and exportation (note that only the routes connecting two different countries regardless of the domestic routes were considered). In this table, airports in the Far East and Middle Asia such as Singapore, Hong Kong, Dubai, and Sharjah display the highest importation values (note that Singapore ranked the first in both categories). Unsurprisingly, major air hubs in Europe (such as airports in London, Paris and Frankfurt) also showed high potential incoming *P. falciparum* flows. Miami is the only airport in the USA on the importation flow top ten lists, with its strong connections to Central and South America. In terms of exportation, the largest airports by traffic capacity and connections to the rest of the malaria-endemic world were highlighted (Additional file 4). Mumbai was ranked first as the largest exporter of *P. falciparum* and *P. vivax* flows, suggesting that it likely acts as an important portal for spreading malaria to the rest of the world.

The betweenness centrality metric was utilized to inspect the connectivity from endemic areas. As the betweenness metric *C*_*B*_(*v*) is defined as the number of shortest paths connecting any two airports that involve a transfer at airport v, high centrality airports in endemic areas provide hubs for people originating at less-accessible airports in remote places to reach the rest of the world. Thus the betweenness centrality presents an initial quantification for the likelihood that an airport routes or spreads malaria flow as a ‘malaria hub’. Table 1 shows the top ten highest betweenness centrality airports for transferring *P. falciparum* flow and *P. vivax* flow elsewhere. For the *P. falciparum* flow, international airports in Africa play important roles as hubs for routing infections. Some airports are observed to have small degrees (low numbers of connecting routes) and large centrality (importance as a hub), which can be considered as an abnormality [47]. These airports connect less accessible and connected airports in endemic areas to other airports in the world. For *P. vivax* flow, Asian international hubs play more important roles. Of interest is Phoenix airport, which ranked the sixth in terms of *P. vivax* centrality, suggesting that it plays an important role as a gateway in linking *P. vivax*-endemic areas to the USA. Additional file 5 presents the spatial distribution of betweenness centrality scores for airports, weighted by *P. falciparum* or *P. vivax* flows.

**Table 1:** **Top ten *Plasmodium falciparum/Plasmodium vivax *betweenness centrality airports with their degrees in a sub-network that only contains direct links from airports in *Plasmodium falciparum- *or *Plasmodium vivax*-endemic areas**

**Top ten *P. falciparum *centrality airports**				
**Airport**	**City**	**Country**	**Normalized betweenness centrality**	**Degree**
NBO	Nairobi	Kenya	47.35	80
MBA	Mombasa	Kenya	32.44	27
JRO	Kilimanjaro	Tanzania	32.39	14
BOM	Mumbai	India	30.41	104
ADD	Addis Ababa	Ethiopia	28.21	64
DEL	Delhi	India	23.16	111
JIB	Djibouti	Djibouti	19.77	15
ADE	Aden	Yemen	18.63	15
MGQ	Mogadishu	Somalia	14.45	8
HRE	Harare	Zimbabwe	14.35	20
**Top ten *P. vivax *centrality airports**				
**Airport**	**City**	**Country**	**Normalized betweenness centrality**	**Degree**
BKK	Bangkok	Thailand	96.43	146
ICN	Seoul	South Korea	78.12	150
DEL	Delhi	India	59.55	133
BOM	Mumbai	India	34.17	116
KMG	Kunming	China	30.79	90
PHX	Phoenix	USA	28.63	91
DPS	Denpasar Bali	Indonesia	27.94	34
SJO	San Jose	Costa Rica	27.72	37
DOH	Doha	Qatar	25.91	100
TAS	Tashkent	Uzbekistan	25.85	69

The betweenness centrality scores show how many shortest paths go through an airport, thus they highlight the potential that airports might route infection flows, acting as ‘malaria hubs’. The degree measures how many routes are linked to an airport.

To further investigate the effects of flows from endemic zones, Additional file 6A and B shows the sums of international incoming risk flows for all the airports in those 36 countries that have national policies for malaria elimination, and are closest to eliminating the disease [55]. Importation of infections threatens the success of elimination programmes [19] and while air travel may not be the highest risk source for these introductions for most of these countries, it remains a potentially important source of incoming infections. From these two maps, it can be seen that China and countries in Middle Asia are subjected to the greatest pressure of incoming flows, relative to other elimination countries, due to their larger incoming traffic volumes from endemic regions elsewhere around the world. To sum up, detailed analyses on airport connectivity are provided in Additional files 2, 3, 4, 5 and 6.

### Connectivity to Southeast Asia

**Figure 2:**
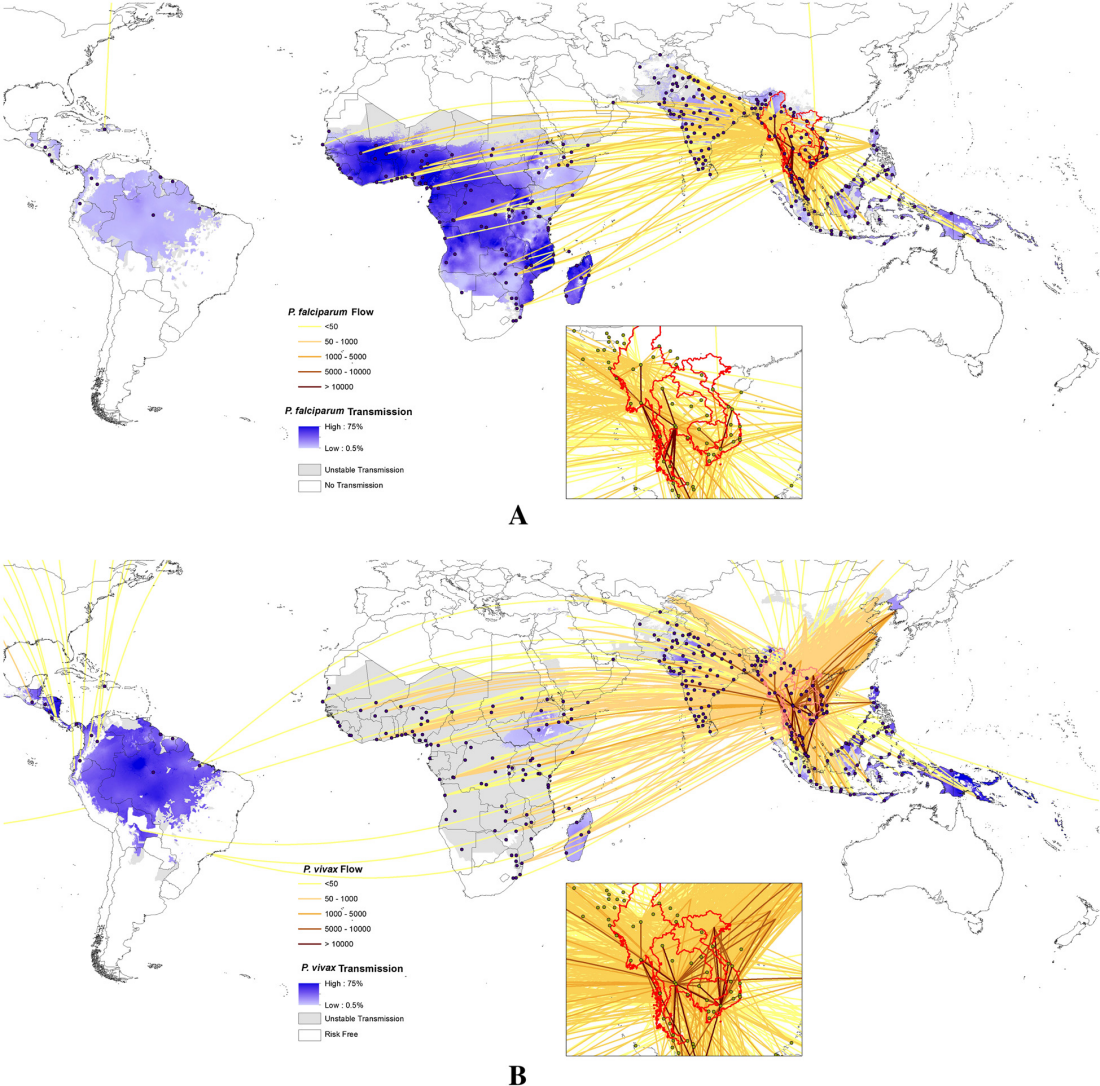
**Estimated relative *Plasmodium falciparum/Plasmodium vivax *flows originating from the Great Mekong subregion overlaid on *Plasmodium falciparum/Plasmodium vivax *prevalence maps. A)***P. falciparum* flows originating from the Great Mekong subregion. **B)***P. vivax* flows originating from the Great Mekong subregion. The flows include estimated passenger numbers, including direct, one-transfer and two-transfer flight routes. The inset maps show close-up views for airports in the Greater Mekong subregion.

Figure 2 maps out the passenger flows scaled by origin prevalence for *P. falciparum* and *P. vivax* from the Greater Mekong subregion. Significant amounts of flow exchange within Southeast Asia can be seen in the close-up subsets. For both *P. falciparum* and *P. vivax* it can be seen that the connectivity, through numbers of travellers, to Latin American endemic regions is weak, but that much stronger connections to sub-Saharan Africa and the Indian subcontinent exist. Increasing connections through trade and labour markets between Asia and Africa over the past decade is exemplified here in the strong connections between the Southeast Asian region and all of sub-Saharan Africa’s major airport hubs. Additional file 7 presents the top ten risk routes spreading drug resistance of *P. falciparum* and *P. vivax* from the Greater Mekong subregion to non-Asian destinations, with estimated *P. falciparum /P. vivax* flow and the number of stops needed to travel from the origin city to the destination city shown.

## Discussion

The continuing growth in air travel is playing an important role in the global epidemiology of malaria. Flight routes now connect previously isolated malaria-endemic regions to the rest of the world, and travellers on these routes can carry infections to the opposite side of the world in less than 24 hours. While many endemic areas still remain relatively isolated, the malaria-endemic world is becoming increasingly connected to both malaria-free areas and other endemic regions. The impacts of this can be seen in imported cases, vector invasions and the spread of drug-resistant parasite strains. Here a spatial network analysis approach were presented to demonstrate the connectivity that exists across the malaria endemic world through air travel, and provide quantitative indicators of the risks it results in for malaria movement.

Results highlight the substantial connectivity that now exists between and from malaria-endemic regions through air travel. While the air network provides connections to previously isolated malarious regions, it is clear that great variations exist, with significant regional communities of airports connected by high rates of prevalence-scaled flow standing out (Figure 1). The structures of these networks are often not geographically coherent, with historical, economic and cultural ties evident. As new routes continue to be established, these communities will likely change, with new popular travel routes, such as those between China and Africa [3] likely altering global malaria flow routes, and new destinations that might encounter increased risks of imported malaria will emerge (Additional file 4 and Table 1). These community maps (Figure 1) and lists of cities by likely import/export of infections (Additional file 4) and hubs for infection flow (Table 1) provide a quantitative picture of how malaria infections are likely moving globally through air travel, and information from which global surveillance strategy design can draw upon. Additional file 4 and Table 1 highlight that certain airports provide significant hubs and gateways for the movement of infections and their entry into countries, and that these are widely distributed across the world. Their role in providing important nodes as both significant through-flow of infections in the network, and entry and exit gateways for cases to/from regions means that they potentially represent valuable sentinel sites for focussed surveillance. Finally, Figure 2 provides a stark reminder of how well connected the malaria-endemic areas of Africa are now to Southeast Asia, illustrating the many possible routes that artemisinin-resistant strains could take. These routes can provide a first-step quantification to support the global plan against artemisinin resistance containment [35] and design of surveillance systems [56], and should be refined with information on the locations of resistance found. Such data could also inform decisions on where and how to limit the risk of spread, for example by pre-travel or arrival screening and treatment.

A range of limitations and uncertainties exist in the analyses presented here. In terms of the quantification of malaria transmission, the use of static maps of annual average prevalence [40,41] neglects the seasonality in transmission that is common to many areas, and also the substantial changes in transmission intensity seen in a variety of locations in recent years [57]. In most parts of the world, the densities of *Anopheles* mosquitoes change seasonally, thus impacting the receptivity of these areas to malaria flows incoming through air travel. While data on changes in *Anopheles* densities globally are not available, temperature-driven models of malaria transmission suitability [43] could be integrated in future work to better account for this and the seasonally varying topologies of the global malaria connectivity network studied. The demographic and behavioural differences between passengers are not accounted for here. Those taking regular air travel are often richer [58], and less likely to be infected, while those that are actually infected and showing symptoms may be less likely to travel. Hence, the air travel passenger dataset used here clearly contains some biases when addressing malaria risks. Further, only parasite prevalence was used as a malaria metric, and while this may be an adequate measure of population prevalence at origin locations, it is not so appropriate for assessing the risk of infection acquisition for naïve travellers, and entomological-based indices are likely more appropriate here, as used in more local studies [27,51,59]. Finally, the examination of relative artemisinin resistance spread risk focuses simply on all travel from four countries, and thus does not account for any heterogeneity in resistance in the region.

Uncertainties and limitations relating to the travel data used also exist. The modelled passenger flows represent just a 2010 snapshot, and thus routes and changes since then are not captured, while inherent uncertainty due to the modelling process also exists [36]. Moreover, the types of traveller and their activities during travel and their residential location are unknown, each of which contributes to differing malaria infection risks. Finally, overland and shipping travel flows are not considered here, which also contribute to local, regional and global malaria connectivity and flows.

This work forms the basis for future analyses on imported malaria, elimination feasibility and the risks and potential routes of artemisinin resistance spread. Rates and routes of imported malaria have been shown to be significantly related to a combination of numbers of travellers to/from endemic destinations and the prevalence of malaria there [3]. The potential thus exists to construct a model based on global malaria prevalence [40,41], the local spatial interaction and accessibility to an airport within a region [60], transmission models for attack rate estimation [27], and traveller flow data [36], that can be used to forecast imported malaria rates, validated with imported malaria data reported by health facilities/organizations.

As nations make progress towards elimination [55], the importance of human movement and imported cases increases. This work contributes to an on-going initiative, the human mobility mapping project [61], aimed at better modelling human and disease mobility, and will form one aspect of continued multimodal assessments of malaria movements [19,27,50,51] and assessment of malaria elimination strategies [23,62]. Finally, the potentially disastrous consequences of the rise and spread of artemisinin resistance requires that detailed and effective planning be implemented in preparation for containing and stemming any spread [56]. A basic assessment here were provided of prevalence-scaled travel from the four Southeast Asian countries where resistance has previously been observed, but significant refinements of these estimates and modelling methods should be undertaken. These may include improved tracking and mapping of observed resistance and human movement patterns in Southeast Asia, as is being undertaken by the TRAC project [63], as well as scenario modelling of the risks of resistance escape to Africa or Latin America. Further, the incorporation of accessibility [64,65] and travel data [51,59] with drug use data (e g, [66]), prevalence information [40,41] and models [67], all undertaken within a probabilistic modelling framework (e. g., [6,60]), could aid in estimation of spread routes should resistance arise elsewhere.

## Competing interests

The authors declare that they have no competing interests.

## Authors’ contributions

ZH and AJT conceived the idea of this analysis. ZH and AJT designed and performed the analysis. ZH and AJT wrote the manuscript. Both authors read and approved the final manuscript.

## Supplementary Material

Additional file 1 **The travel time/distance mask to extract the estimated typical maximum prevalence of *Plasmodium falciparum/Plasmodium vivax* at the origin of travellers.** Inset map: travel time to the nearest major settlement (population size >50,000). The global map of accessibility is obtained at [44]). Main map: each dot shows an airport location with a 50-km buffer around it, and the colours show the global *P. falciparum* prevalence map [40] masked by the global travel time map with a threshold value of less than two hours. These two-hour and 50-km thresholds were used to assign prevalence values to airports (see main text). Click here for file

Additional file 2 **Air travel network communities weighted by directed estimates of passenger flow.** Airports with the same community membership (indicated by the same colour) display stronger links in terms of likely movement volume between them than to airports in other communities. The movement volume is extracted from Huang *et al*’s [36] modelled passenger flow matrix. Click here for file

Additional file 3 **Communities for all possible connections originating from *Plasmodium falciparum/Plasmodium vivax*-endemic areas. A)***P. falciparum* multilevel membership; **B)***P. vivax* multilevel membership. These two maps show directly connected, one-transfer and two-transfer airports from endemic areas. The inset maps present close-up views of the USA and western Europe. Airports with the same community membership (indicated by the same colour) display stronger links in terms of likely movements of infections between them than to airports in other communities. Note that in the *P. vivax* map, two communities with less than ten airports are not shown. Click here for file

Additional file 4 **Top ten airports based on estimated relative malaria importation and exportation rates.***P. falciparum*/*P. vivax* flow measures are calculated based on the incoming and outgoing numbers of passengers travelling internationally, scaled by the malaria prevalence at the origin of the routes in the case of importation, and at the airport listed in the case of exportation. The flows represent a relative measure of infection movement and are not designed to represent actual number of infections. Click here for file

Additional file 5 **Spatial distributions of airports with *Plasmodium falciparum/Plasmodium vivax *betweenness centrality scores. A)** Airports with normalized betweenness scores >0 from *P. falciparum*-endemic areas, weighted by the *P. falciparum*-prevalence weighted passenger flows. **B)** Airports with normalized betweenness scores from *P. vivax*-endemic areas, weighted by the *P. vivax* prevalence-weighted passenger flows. Details on the betweenness metric are provided in the main manuscript. Click here for file

Additional file 6 **Spatial distributions of airport nodes in elimination countries **[55]** weighted by incoming international *Plasmodium falciparum/Plasmodium vivax* flows. A)** Airports in countries with elimination objectives shown with dot size scaled to match total incoming passenger flow weighted by the *P. falciparum* prevalence at the traveller origins. **B)** Airports in countries with elimination objectives shown with dot size scaled to match total incoming passenger flow weighted by the *P. vivax* prevalence at the traveller origins. These two figures highlight the relative risks of infection importations through air travel for each country with malaria elimination objectives [55]. Click here for file

Additional file 7 **Top ten *Plasmodium falciparum* routes and *Plasmodium vivax* routes to non-Asian destinations from the Great Mekong subregion, as defined by malaria-prevalence scaled passenger numbers.** Values with (*) are returned as the adjusted largest flows between two connection flights. Click here for file
