# Supplementary figures and images for: Global malaria connectivity through air travel
Source: Malar J. 2013 Aug 2;12:269. doi: 10.1186/1475-2875-12-269 (PMC3766274; doi:10.1186/1475-2875-12-269)

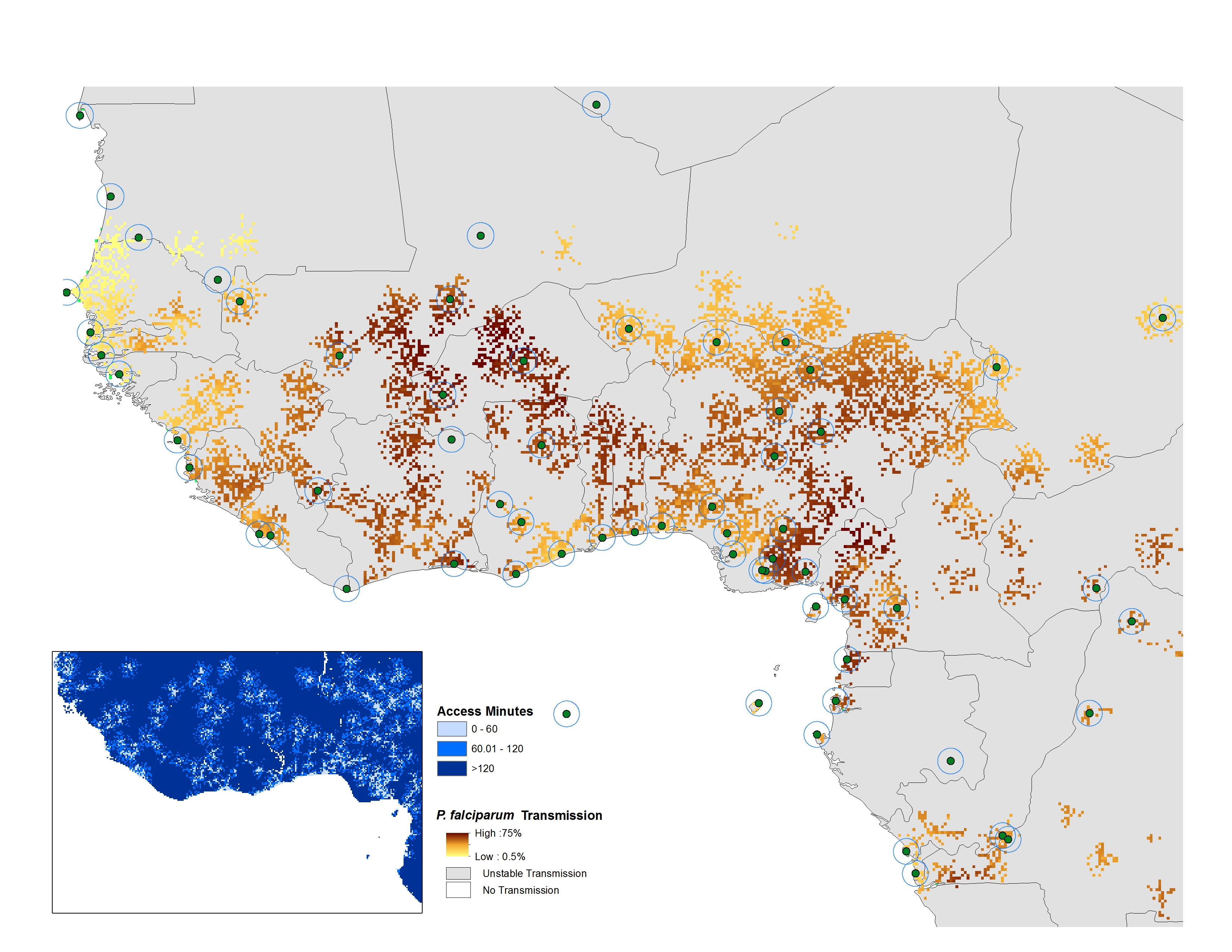

Supplement: Additional file 1 — The travel time/distance mask to extract the estimated typical maximum prevalence of Plasmodium falciparum/Plasmodium vivax at the origin of travellers. Inset map: travel time to the nearest major settlement (population size >50,000). The global map of accessibility is obtained at [44]). Main map: each dot shows an airport location with a 50-km buffer around it, and the colours show the global P. falciparum prevalence map [40] masked by the global travel time map with a threshold value of less than two hours. These two-hour and 50-km thresholds were used to assign prevalence values to airports (see main text). [file 1475-2875-12-269-S1.jpeg]

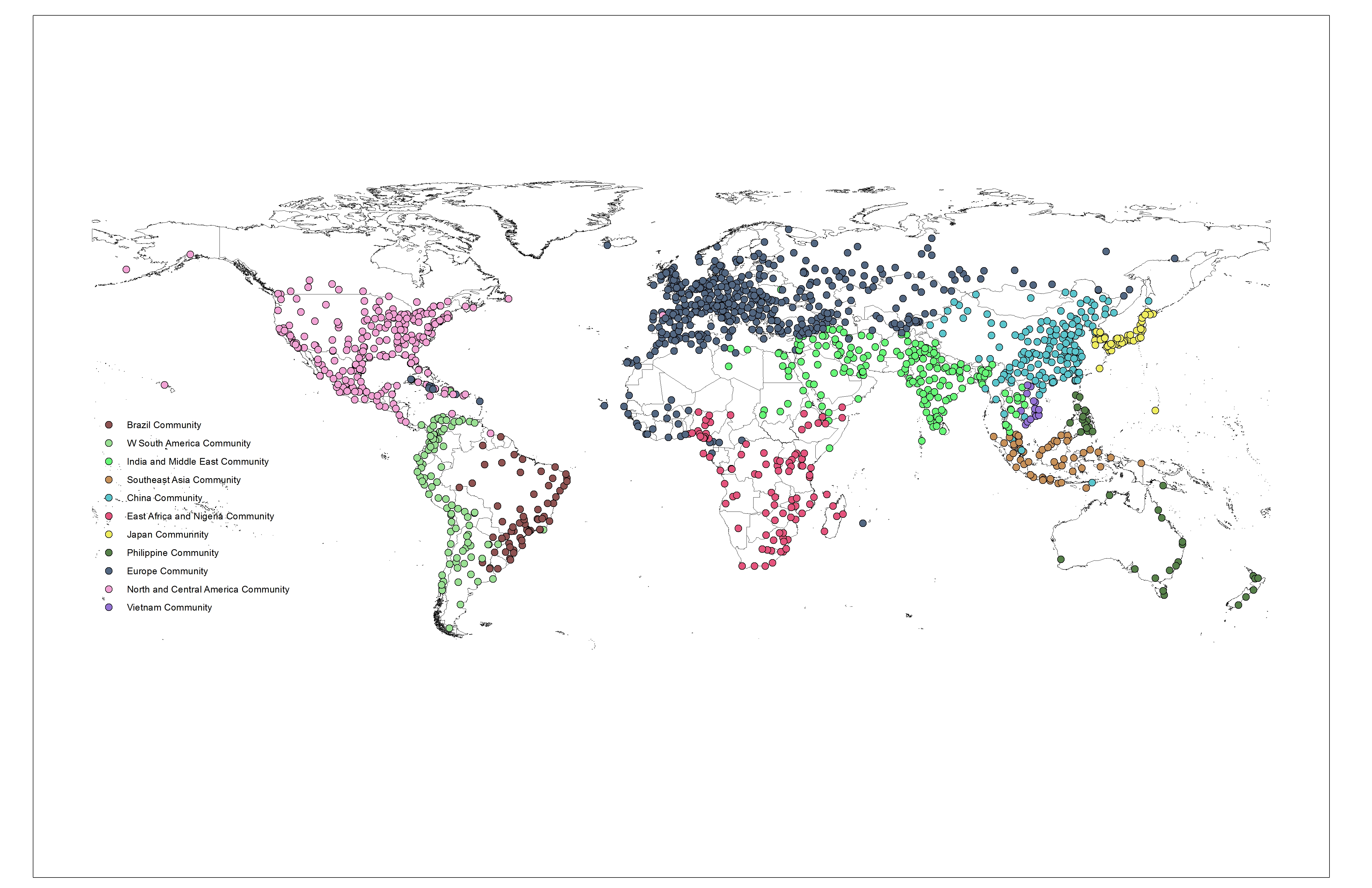

Supplement: Additional file 2 — Air travel network communities weighted by directed estimates of passenger flow. Airports with the same community membership (indicated by the same colour) display stronger links in terms of likely movement volume between them than to airports in other communities. The movement volume is extracted from Huang et al’s [36] modelled passenger flow matrix. [file 1475-2875-12-269-S2.jpeg]

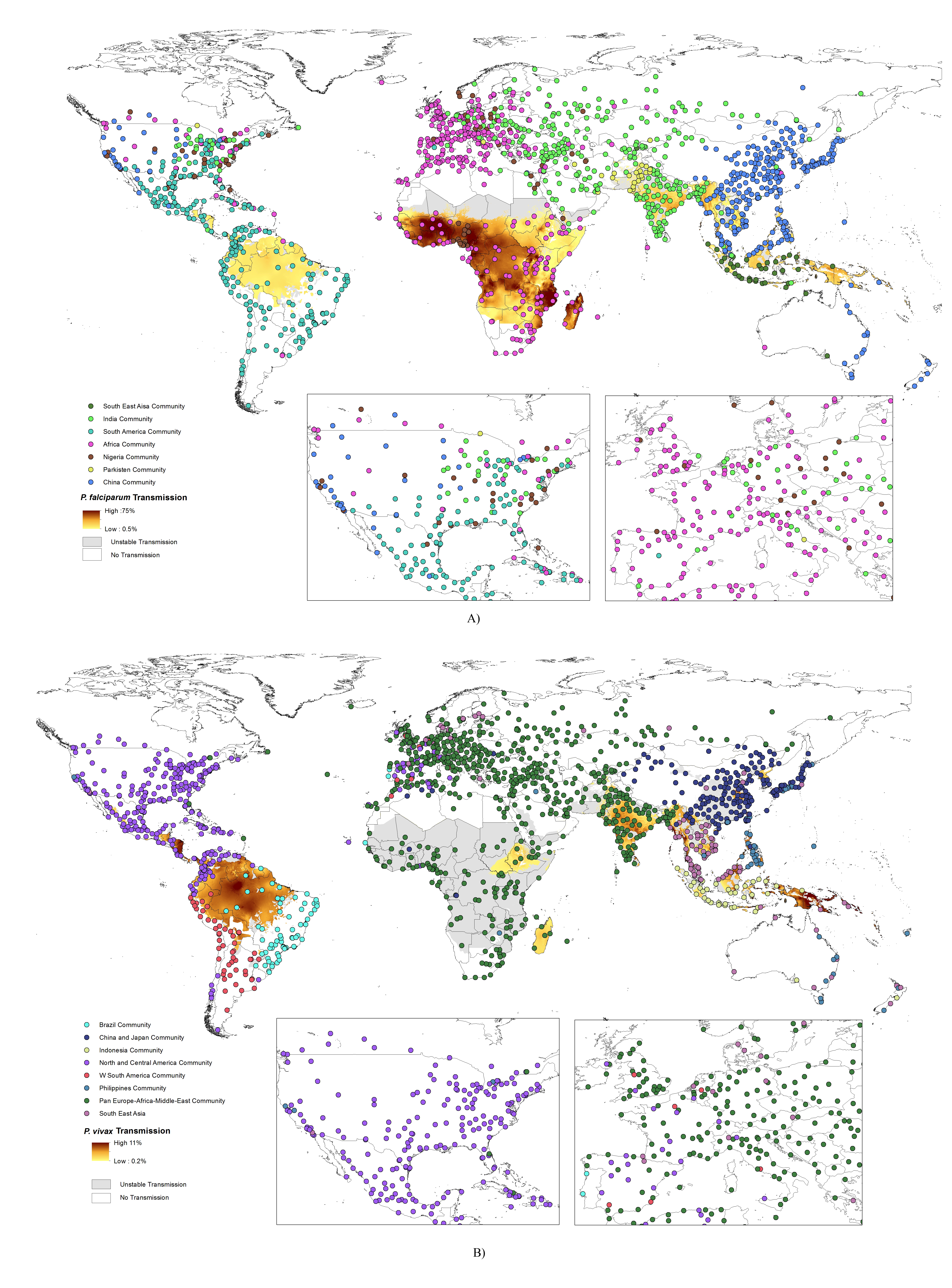

Supplement: Additional file 3 — Communities for all possible connections originating from Plasmodium falciparum/Plasmodium vivax-endemic areas. A)P. falciparum multilevel membership; B)P. vivax multilevel membership. These two maps show directly connected, one-transfer and two-transfer airports from endemic areas. The inset maps present close-up views of the USA and western Europe. Airports with the same community membership (indicated by the same colour) display stronger links in terms of likely movements of infections between them than to airports in other communities. Note that in the P. vivax map, two communities with less than ten airports are not shown. [file 1475-2875-12-269-S3.jpeg]

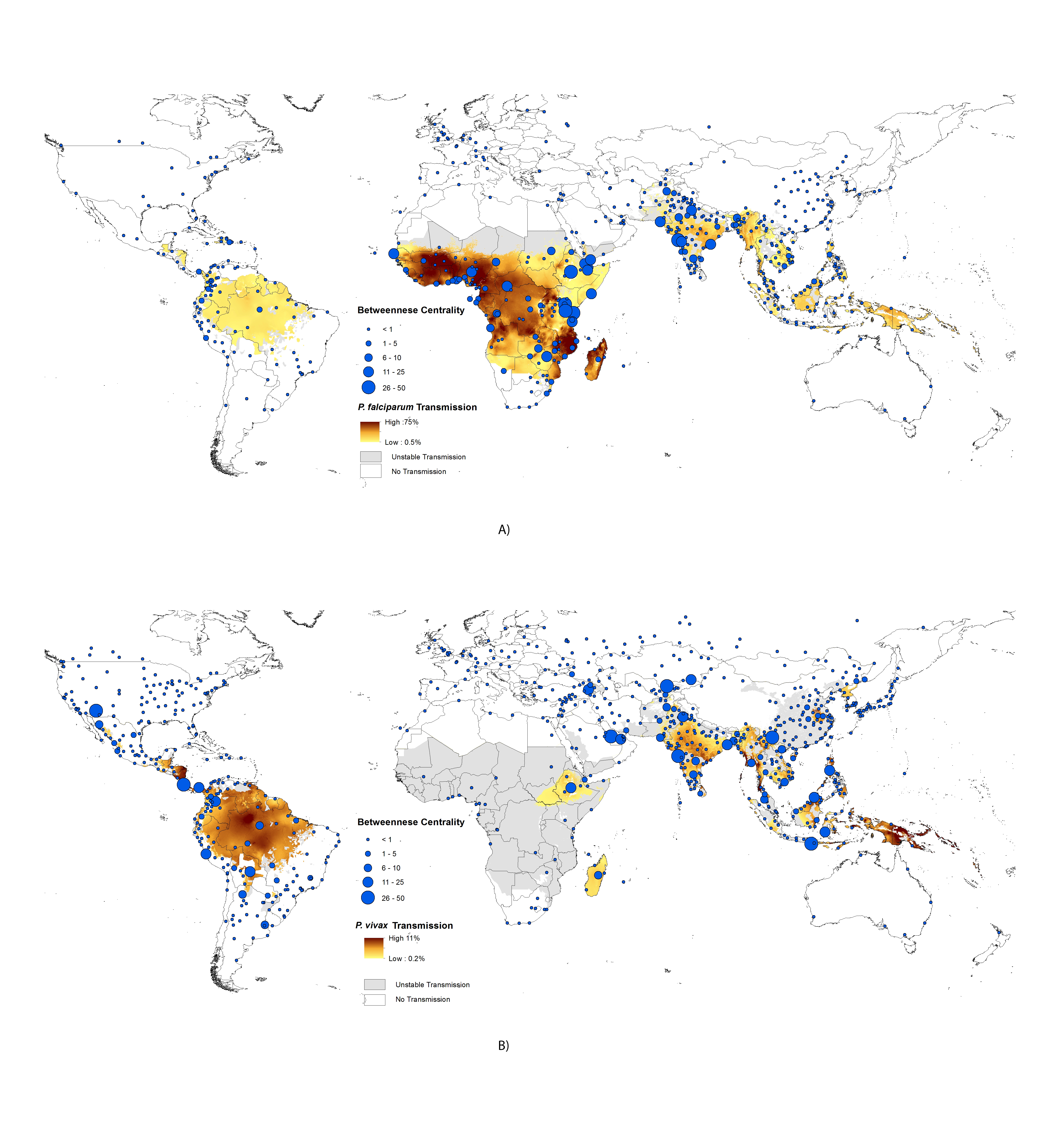

Supplement: Additional file 5 — Spatial distributions of airports with Plasmodium falciparum/Plasmodium vivax betweenness centrality scores. A) Airports with normalized betweenness scores >0 from P. falciparum-endemic areas, weighted by the P. falciparum-prevalence weighted passenger flows. B) Airports with normalized betweenness scores from P. vivax-endemic areas, weighted by the P. vivax prevalence-weighted passenger flows. Details on the betweenness metric are provided in the main manuscript. [file 1475-2875-12-269-S5.jpeg]

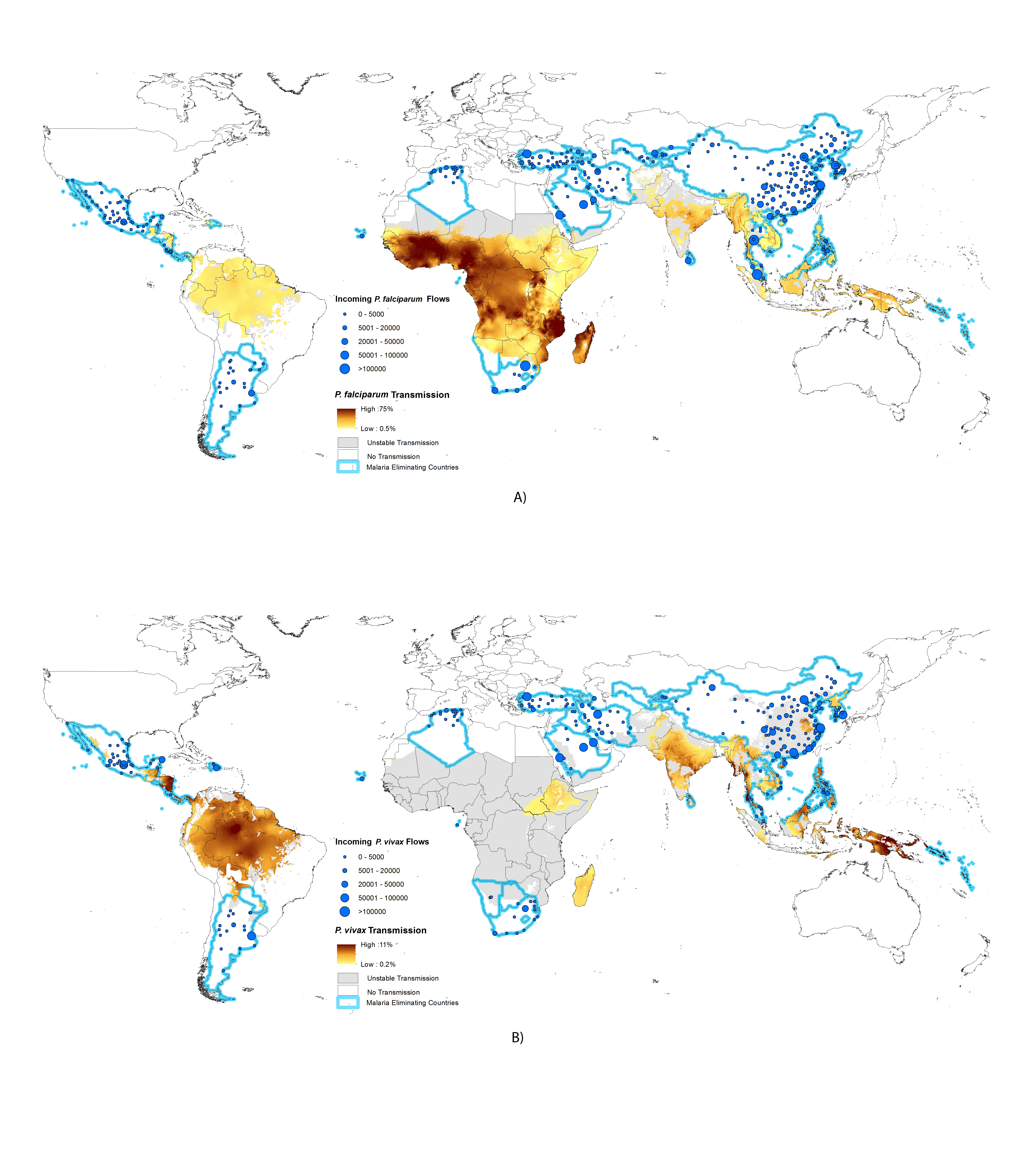

Supplement: Additional file 6 — Spatial distributions of airport nodes in elimination countries [55] weighted by incoming international Plasmodium falciparum/Plasmodium vivax flows. A) Airports in countries with elimination objectives shown with dot size scaled to match total incoming passenger flow weighted by the P. falciparum prevalence at the traveller origins. B) Airports in countries with elimination objectives shown with dot size scaled to match total incoming passenger flow weighted by the P. vivax prevalence at the traveller origins. These two figures highlight the relative risks of infection importations through air travel for each country with malaria elimination objectives [55]. [file 1475-2875-12-269-S6.jpeg]
